# Experimental investigation on enhancing the mechanical properties of biodegradable Zn-3Mg alloys reinforced with snail-shell particulates via powder metallurgy

**DOI:** 10.1007/s10856-025-06963-7

**Published:** 2025-11-22

**Authors:** Maruf Yinka Kolawole, Sana Anwar, Esra Bozkaya, Asli Gunay Bulutsuz, Siyami Karahan, Hakan Yilmazer, Farasat Iqbal

**Affiliations:** 1 Mechanical Engineering Department, Kwara State University, Malete, Kwara State Nigeria; 2 Interdisciplinary Research Centre in Biomedical Materials, COMSATS University Islamabad, Lahore Campus, Defence Road off Raiwind Road, Lahore, Pakistan; 3 Hacılar Vocational School Occupational Health and Safety Program, Kirikkale University, Kırıkkale, Türkiye; 4 Department of Mechanical Engineering, Yildiz Technical University, Besiktas, Istanbul Türkiye; 5 Health Biotechnology Joint Research and Application Center of Excellence, Istanbul, Türkiye; 6 Department of Histology & amp; Embryology, Faculty of Veterinary Medicine, Kirikkale University, Kirikkale, Türkiye; 7 Department of Metallurgical and Materials Engineering, Yildiz Technical University, Istanbul, Türkiye

**Keywords:** Biodegradable Metals, Biodegradable Zn-alloys, Mechanical Properties, Biodegradable Implants, Metal Matrix Composites, Biomaterials

## Abstract

Biodegradable zinc-based alloys are promising candidates as a new generation implant materials due to their favorable degradation rates compared to magnesium and iron. However, their relatively low mechanical strength hinders their clinical usage. In this experimental study, Zn–3Mg/xSnS (*x* = 0.5–6 wt%) composites were manufactured via powder metallurgy. The performance of the obtained samples was systematically investigated via microstructural analysis (SEM), mechanical properties (compressive yield strength, elastic modulus, and hardness), in vitro degradation, and cytocompatibility with L929 fibroblast cells. According to the obtained results, SnS reinforcement significantly improved mechanical performance. Microstructural investigation revealed homogeneous SnS distribution and refinement of intermetallic phases. Among all the sample groups, Zn–3Mg–2SnS resulted in a compressive yield strength of 402 MPa, elastic modulus of 49 GPa, and hardness of 151 HV. Degradation tests were performed for 28 days, and the samples exhibited a moderate corrosion rate ( ~ 0.2 mm/year). Cytotoxicity assays confirmed >70% cell viability at 50% extract concentrations. These results show that Zn–3Mg alloys can be efficiently reinforced with bio-derived SnS particles, improving their strength and biocompatibility without decreasing their degradation performance.

**Graphical Abstract:**

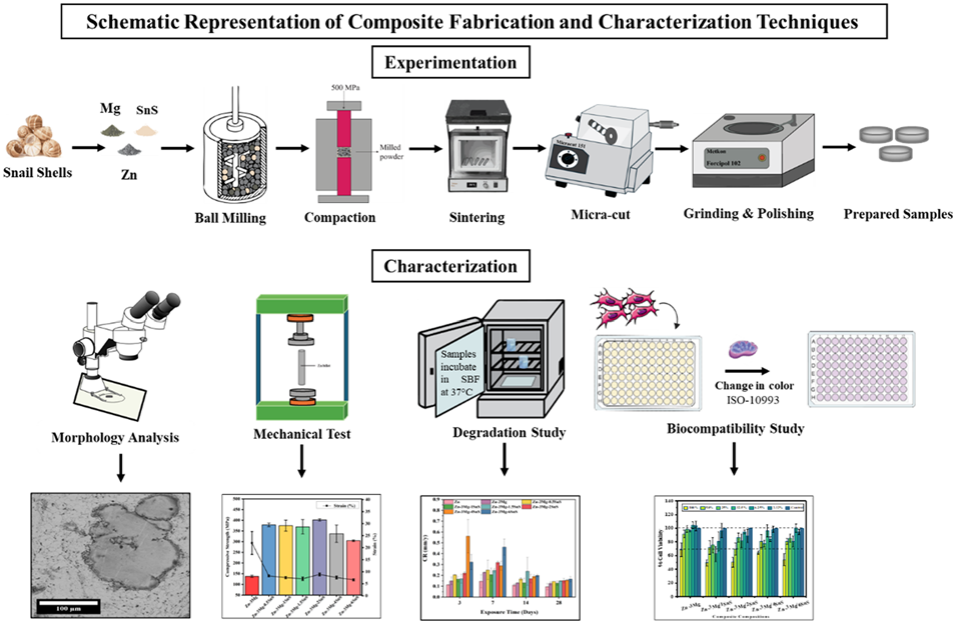

## Introduction

Traditional metallic materials, such as cobalt-chromium-molybdenum alloys, stainless steel, and titanium alloys, are widely used for orthopedic applications. Their non-biodegradability, however, presents serious clinical problems and frequently calls for implant removal following bone healing, raising the risk, expense, and discomfort of surgery [1, 2]. Furthermore, the traditional metallic implant has a relatively high elastic modulus ( > 200 GPa), which is higher than natural bone ( ≤ 45 GPa), leading to stress shielding where the implant bears most of the load, thereby impairing bone remodelling and regeneration. By combining bone support with gradual bio-absorption, biodegradable metallic implants offer a promising substitute that encourages tissue regeneration and natural healing [3, 4]. As stated by Liu et al. and Yang et al. [5, 6] biodegradable metallic implants degrade in vivo and are metabolized by the body. The released corrosion products are harmless to host tissues [5, 6]. These implants provide temporary mechanical support, thereby avoiding the long-term complications associated with permanent implants. [7, 8]. Moreover, biodegradable metals such as zinc and magnesium have an elastic modulus closer to that of natural bone, which helps reduce mechanical mismatch between the implant and bone. Owing to these attributes, biodegradable metals are being extensively explored for orthopedic and cardiovascular applications [9]. Magnesium, iron, and zinc are the three principal biodegradable metals investigated for biomedical applications [8, 10]. However, each exhibits specific limitations that hinder clinical translation. Magnesium alloys, despite their favorable mechanical strength, have a corrosion rate that is too high leading to hydrogen evolution, excessive ion release, and premature loss of mechanical integrity [11–16]. On the other hand, iron corrodes too slowly for timely resorption in orthopedic applications. Beyond these, zinc demonstrates an intermediate and more suitable degradation rate, but its poor mechanical strength in pure form restricts its use [17, 18]. To address these challenges, researchers are developing Biodegradable Metal Matrix Composites (BMMCs), in which zinc or other base metals are reinforced with biocompatible, bioactive phases. With the reinforcement effect in the composites, mechanical performance can be enhanced with optimized corrosion behavior and improved biological interactions [5, 19, 20]. Among other BMMCs, Biodegradable Zinc Matrix Composites (BZMCs) are popular due to zinc’s biocompatibility and favorable corrosion rates. In previous studies, Hydroxyapatite (HA) reinforcement was added to the zinc matrix through spark plasma sintering, which significantly improved mechanical strength, making the composites suitable for both cancellous and cortical bone applications [20, 21]. Besides the positive effect of additives on the mechanical properties, the degradation rate of these composites was found to be twice ( ≈ 0.4 mm/y) that of pure zinc ( ≈ 0.2 mm/y), which is advantageous for scaffolding and cancellous bone augmentation [22, 23]. These studies highlight the potential of BZMCs as a new material for the new generation of biodegradable orthopedic implant applications.

Besides pure Zn, Mg alloys, such as Zn–3Mg [24], were also studied for orthopedic applications due to its balanced combination of strength, ductility, and corrosion resistance. The addition of Mg to the system enhanced hardness and fatigue resistance compared with pure zinc and lower-Mg alloys, suggesting suitability for long-term implant performance. However, our previous work on powder-metallurgy (PM) processed alloys showed that while Zn–2Mg achieved a maximum ultimate tensile strength (UTS) of 210 MPa, the UTS of Zn–3Mg decreased to 110 MPa, indicating that higher Mg contents may compromise strength despite improving biodegradability and bioactivity [25]. From this point of view, Zn–3Mg was selected in the present study as the base alloy to investigate whether reinforcement with sintered snail shell (SnS) particulates can enhance its mechanical and biological performance. Snail shells are low-cost, and they have high calcium carbonate content ( ≈ 81% CaCO₃). Recent reports have demonstrated their potential as a calcium source for hydroxyapatite synthesis, highlighting the potential of snail shells for enhancing the biocompatibility of zinc-based biomaterials in biomedical applications [26–29].

To produce biomechanically compatible composites with improved biological responses and high densities, a variety of manufacturing techniques, such as hot pressing, melt casting, in situ alloying, and spark plasma sintering, have been used as shown in Table 1. For these reasons, in this experimental study, the PM route was chosen to ensure a homogenous dispersion of reinforcements within the Zn–3Mg matrix for microstructural homogeneity and property reproducibility. To the best of our knowledge, no previous research has reported the use of bio-derived SnS particulates as reinforcement in Zn–Mg based alloy systems. The development and characterization of SnS particulate-reinforced Zn–3Mg composites were the main objectives of this study. Their mechanical performance, degradation rate, and cytocompatibility were investigated to understand the potential of the target composite for orthopedic implant applications.

**Table 1:** Effect of processing route and reinforcement type on Zn alloys

Sr #	Base	Reinforcement type	Methods	Key findings	References
1	Zn	Hydroxyapatite	SPS	Tunable degradability, improved osteoconductivity	[38]
2	Zn	Graphene nanosheets	SPS	Accelerated degradability, improved interface bonding	[53]
3	Zn	Monetite	SPS	Improved ductility	[54]
4	Zn	Mg_2_Ge	In situ alloying	Improved mechanical properties, moderate corrosion rate (CR)	[55]
5	Zn	ZnO composites	Melt casting	Enhanced mechanical property, moderate biocompatibility	[56]
6	Zn	Mg-Si	PM	Improved wear resistance	[57]
7	Zn	Mg	PM	Reduced porosity through hot pressing	[58, 59]

## Materials and methods

Pure zinc (Zn: Sigma-Aldrich chemicals, Pakistan, 99.8% purity, 44 µm) and magnesium (99% purity, 100 µm) powders were obtained from Duksan products, Comsats Lahore, Pakistan. Snail shells, sourced from Abegunde Village, Okitipupa, Ondo State, Nigeria, were washed, oven-dried, pulverized, and calcined at 900 °C in air for 3 h [29]. The resulting calcined powder was then blended and sieved to obtain a particle size of 63 µm and was used as reinforcement material in this work.

### Zn-3Mg alloy and Zn-3Mg/xSnS composites production

Zn-3Mg alloy and its composites were synthesized using powder metallurgy. Measured quantities of Zn-3Mg alloy powder and Zn-3Mg/xSnS mixtures (where *x* = 0, 0.5, 1.0, 1.5, 2, 4, and 6 wt%) were weighed and mixed in a planetary ball milling machine at 300 rpm for 3 h. The ball-to-powder ratio (B:P) was 10:1, following previous studies [30, 31]. No lubricant or additives were used during mixing.

**Fig. 1:**
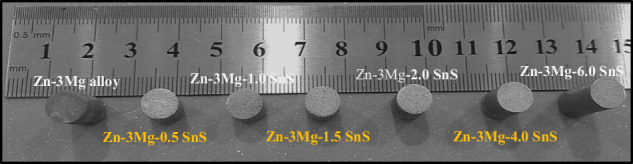
Sintered billets of Zn-3Mg-xSnS composites

Before powder compaction, the die wall and punch surfaces were lubricated with a slurry of zinc stearate (Sigma-Aldrich) and silicon oil (1:1). The milled powders were then pressed into pellets under 500 MPa for 180 s using a 25-ton capacity auto-series Caver hydraulic press. The green pellets, as shown in Fig. 1, were stored in airtight vials within a desiccator before sintering.

**Fig. 2:**
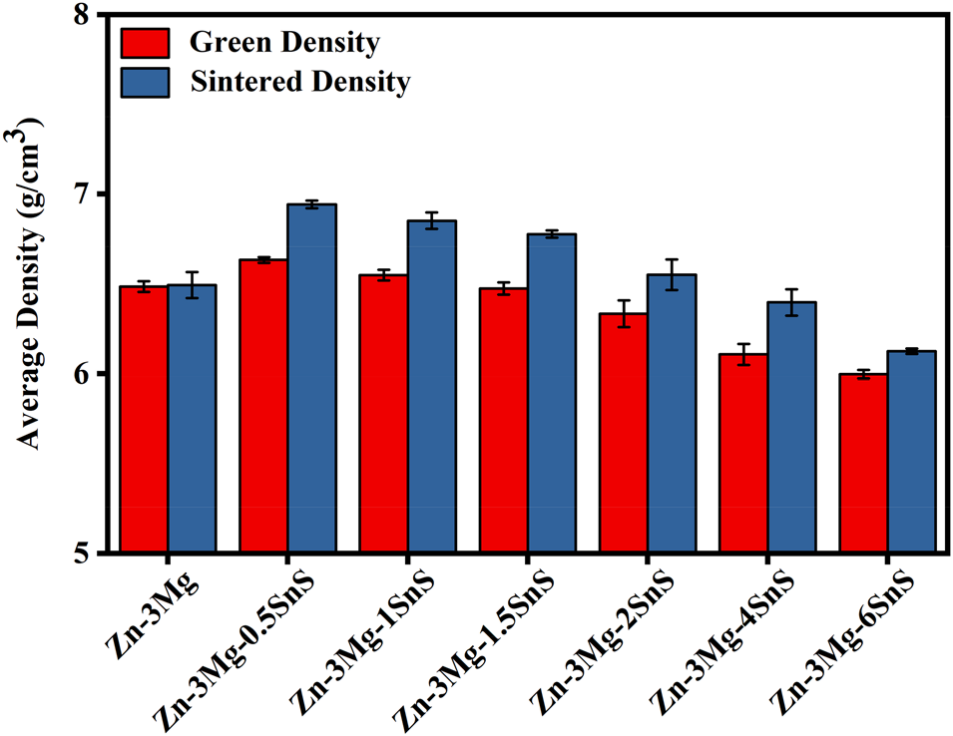
Density of sintered Zn-3Mg/xSnS billets with increasing wt% SnS addition

The green samples were de-lubricated in a Vulcan electric furnace at 200 °C with a heating rate of 10 °C/min for 4 h of soaking time. This de-lubrication step was performed before the actual sintering process. Zn-3Mg/xSnS composites were then sintered at 350 °C using a Qex Centurium vacuum furnace with a heating rate of 20 °C/min and a holding time of 4 h. Finally, the sintered samples, as shown in Fig. 2, were quenched in stagnant water to prevent excessive grain growth. During characterization, each measurement was performed in triplicate, and mean values were used for statistical analysis. The overall experimental workflow is illustrated in the graphical abstract for clarity.

### Green and sintered densities measurements

The green and sintered densities of Zn-3Mg alloy and Zn-3Mg/xSnS composites were measured using Archimedes’ principle according to ASTM B962-17. The weight ($$w)$$ in air was measured using a digital weighing balance. The dimensions of the samples were measured using a Vernier caliper to determine the total volume ($$V$$). The apparent green ($${\rho }_{g})$$ and sintered densities ($${\rho }_{s})$$ were evaluated using Eq. (1) according to Tahmasebifar et al. [32]. For the sake of statistical reliability, the procedure was repeated five times.

1 $$p=\frac{w}{v}$$

### Microstructure and phase assessment

Microstructures of Zn-3Mg and Zn-3Mg/xSnS composites were examined using a TESCAN Vega3-LMU Scanning Electron Microscopy (SEM) equipped with energy dispersive spectroscopy from Oxford Instruments Inc, UK. The SEM was operated at 20 kV for imaging. Before SEM analysis, samples (8 mm diameter, 2 mm height) were prepared using a micro cut machine, followed by grinding with 2000 grit sandpaper under running water for continuous cooling. The samples were then cleaned ultrasonically in ethanol and distilled water for 15 min. Finally, the samples were etched in a solution containing 2% HNO_3_ and 95% ethanol according to Li et al. [33] for SEM examination. The phase identifications of the prepared sintered samples were done by means of X-ray diffraction (XRD) using Cu-kα radiation (wavelength: $\lambda =$ 1.5406 A˚) generated at 40 kV and 30 mA at 0.02° step size with 1–5 s per step over a 2θ angular range of 5–100°. The phase identification was analyzed using X’Pert HighScore software.

### Mechanical testing

The microhardness of the Zn-3Mg-xSnS composites was evaluated on a microhardness tester (No: HVS1000 with incorporated triboscope), following ASTM-E384-17 standard [31, 34]. Samples (8 mm diameter, 3 mm height) were ground up to 1200 grit size using SiC abrasive paper to obtain a silvery smooth surface. The microhardness measurement involved placing the sample on the stage, focusing on the desired location, and applying a 100 kgf load for a dwell time of 10 s. The average hardness obtained from five indentations was recorded as the sample hardness.

Compressive strength and elastic moduli were determined in accordance with the ASTM E9-09 standard procedure. Three samples per variant of the Zn-3Mg alloy and Zn-3Mg/xSnS composites were tested at a strain rate of 10^−3^s^−1^ on a Universal testing machine (Testometric TUE-C-500, UK model 50 kN capacity) [35]. All samples maintained a constant length-to-diameter ratio (l/d = 2). The elastic modulus of each sample was calculated from the corresponding compressive strength data.

### Immersion test

Before immersion, the samples were ground using SiC papers up to #4000 grit and polished to a mirror-like finish. Afterwards, the samples were cleaned in ethanol using ultrasonic cleaning and dried with cool air. The surface area-to-volume ratio of the sample to SBF was set at 1 cm²:30 mL. Samples were placed inside an inverted syringe, and hydrogen bubbles generated during immersion were collected in the syringe cap. The volume of hydrogen gas was determined by measuring the liquid displacement within the syringe, allowing for real-time monitoring of the corrosion rate of Zn-3Mg-SnS. The system was maintained at 37 °C to simulate body temperature.

After a predetermined period (28 days), samples were removed, gently rinsed with deionized water, and air-dried. The corrosion morphology and composition of the corrosion products were analyzed using SEM. The corrosion rate (mm/y) was calculated using the weight-loss method. Weight loss was determined by measuring the samples’ weights before and after immersion, and the average of three measurements was taken for each sample group.

### Cytocompatibility study

The cytotoxicity of the Zn-3Mg and Zn-3Mg/xSnS composites was assessed using MTT assay following ISO 10993-5 standard. This colorimetric method utilizes 3-(4,5-dimethylthiazol-2-yl)-2,5-diphenyltetrazolium bromide (MTT) to evaluate cell proliferation. L929 fibroblast cells were seeded into a 96-well plate at a density of 1.0 × 10^4^ cells per well and cultured for 24 h (37 °C, 5% CO_2_). The cells were then exposed to the different extract concentrations prepared according to the TS EN ISO 10993-12. Both the diluted extracts and the cells were incubated together for 24 h (37 °C, 5% CO_2_) with six replicates per sample. The positive and negative controls, as well as blank samples containing only the culture medium, were run in triplicate. After incubation, the culture media were removed, and 50 μl MTT (1 mg/mL) was added to each well. Following a further incubation at 37 °C for 2 h, 100 µl of isopropanol was added to each well. The absorbance of the resulting solution was measured at 570 nm using a microplate reader (SpectroSTARNano Microplate Reader, BMG LABTECH, Germany). The cell viability was then calculated relative to the blank sample as shown below:

$$\% {\rm{Cell\; Viability}}=\frac{100* {\rm{OD}}570{\rm{e}}}{{\rm{OD}}570{\rm{b}}}$$

Where OD570e: is the mean value of the measured optical density of the 100% extracts of the test sample, and OD570b: is the mean value of the measured optical density of the blanks.

### Statistical analysis

Triplicate samples were used for each time interval to ensure repeatability. The p-value of two-way ANOVA of variance < 0.05 was applied on the degradation and cytocompatibility test to evaluate the significant difference in data.

## Results and discussion

### Green and sintered density of Zn-3Mg-xSnS composites

The density results of the green and sintered densities of the Zn-3Mg alloy and Zn-3Mg/xSnS composites are given in Fig. 2. An increase is observed for Zn-3Mg alloy with sintering from 6.485 g/cm³ to 6.493 g/cm³. With the addition of 0.5 wt% SnS the density increased more (4.66%) after sintering, with similar improvements observed up to 1 wt% SnS. In contrast, at higher percentages of SnS, the densification performance decreased. The densification ratio decreased from 4.68% for Zn-3Mg/0.5SnS to 2.12% for Zn-3Mg/6SnS, which shows that the effectiveness of sintering decreased with increasing amount of reinforcement. The addition of SnS decreased density in all groups because of their lower density (1.63 g/cm³) compared to pure Zn (7.13 g/cm³). Moreover, the presence of micro-pores and possible wettability problems between the matrix and the reinforcement may hinder densification during sintering [36].

These observations are consistent with previous reports by Soon et al. [37] and Yang et al. [38], who also observed a decrease in relative density when hydroxyapatite was introduced into Mg-Zn alloys and Zn matrices, respectively.

### Microstructure and phase analysis of Zn-3Mg-xSnS composites

**Fig. 3:**
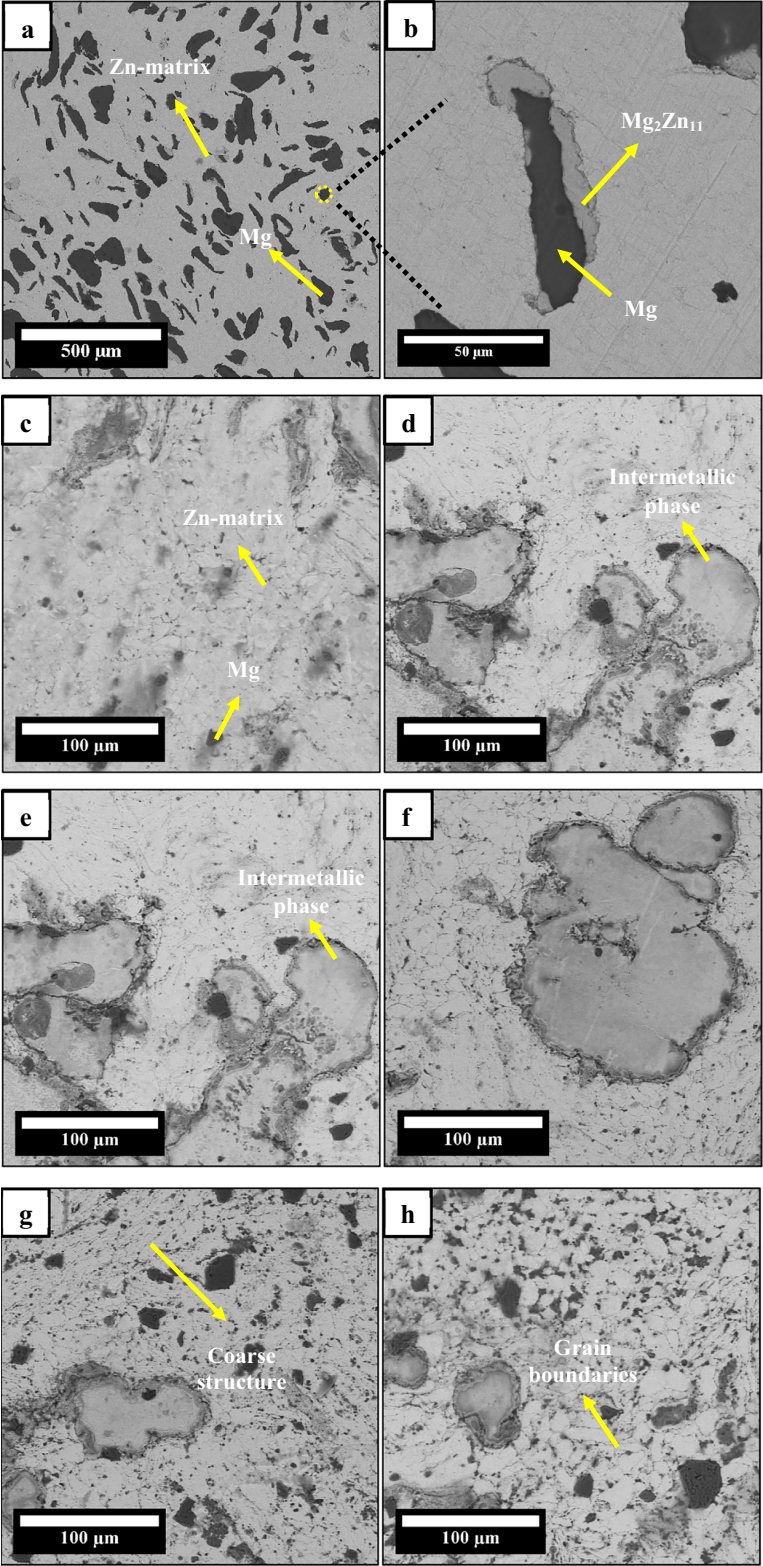
SEM images of **a** Zn-3Mg (500 µm) **b** Zn-3Mg (50 µm) **c** Zn-3Mg-0.5SnS **d** Zn-3Mg-1SnS **e** Zn-3Mg-1.5SnS **f** Zn-3Mg-2SnS **g** Zn-3Mg-4SnS **h** Zn-3Mg-6SnS

SEM images of the sintered Zn-3Mg/xSnS alloy and composites are given in Fig. 3. In Fig. 3a, for the Zn-3Mg intermetallic phases, an α-Zn-rich phase and large Mg-rich lump regions were observed. The presence of pores at the Zn-3Mg interface indicates poor interfacial bonding because of the limited diffusion of magnesium into the zinc matrix. Moreover, the coarse, non-precipitated Mg-rich phases could reduce dislocation density at the interface, thereby weakening the alloy’s mechanical strength and integrity. Notable microstructural changes were observed with the addition of SnS reinforcement. In Fig. 3b, for the 0.5 wt% SnS sample, a more uniform distribution of fine-grained intermetallic, eutectoid, and α-Mg-rich phases was observed, along with refined grain sizes and improved interfacial bonding. This behavior was similar up to 1.5 wt% SnS (Fig. 3c–e). Only a few Mg grains were visible, suggesting that the addition of SnS as reinforcement promoted Mg precipitation and acted as a grain refiner. In the Zn-3Mg/2SnS composite (Fig. 3d), the Mg-rich phase was completely transformed into fine intermetallic and α-Zn phases, a morphology expected to increase dislocation density and enhance mechanical properties. However, at higher SnS contents (Fig. 3g, h), grain coarsening of intermetallic phases and the appearance of open pores were evident. These results can act as stress concentrators, which can reduce the mechanical performance of the Zn-3Mg/SnS composites, especially at increased reinforcement levels [39].

**Fig. 4:**
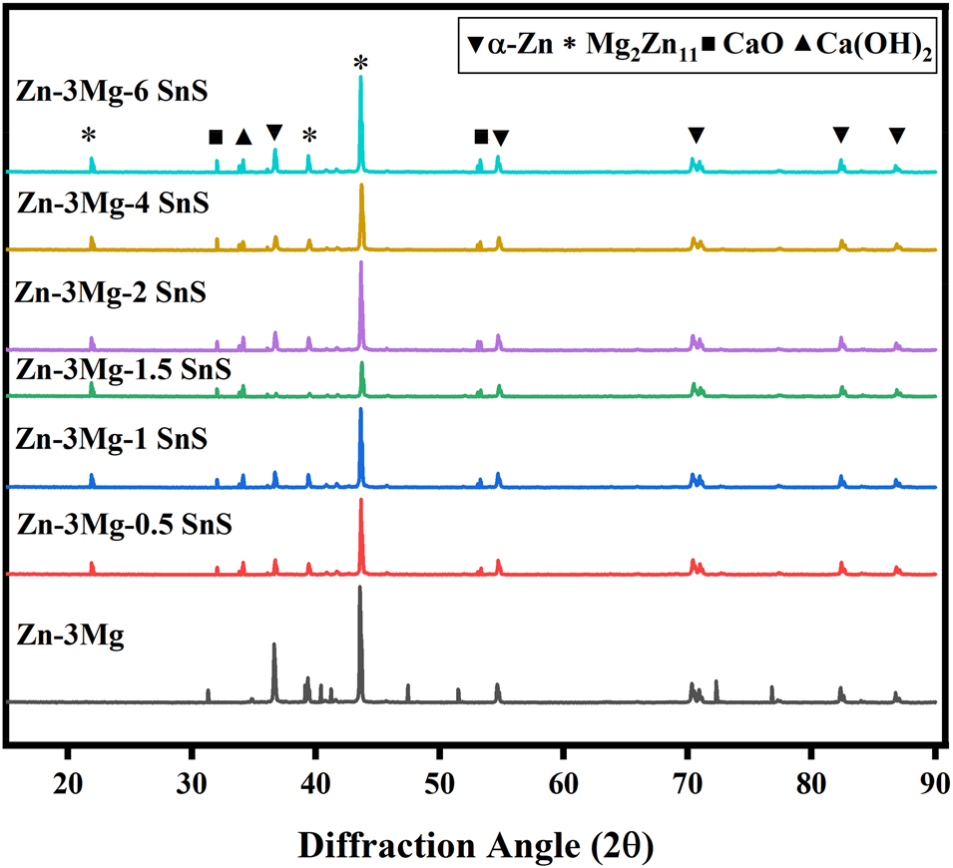
XRD spectra of pure Zn, Zn-3Mg alloy and Zn-3Mg/xSnS composites

XRD analysis of the sintered Zn-3Mg alloy and Zn-3Mg/SnS composites are given in Fig. 4. The results indicate that α-Zn was the dominant phase and the intermetallic was Mg₂Zn₁₁, as shown in Fig. 4. In addition, minor phases of calcium oxide and calcium hydroxide were detected at 32.01° and 53.59° (2θ) for CaO, and 36.74° (2θ) for Ca(OH)₂. These two minor phases belong to the snail shell-derived precursor: the calcium carbonate (CaCO₃) present in the shells decomposed during high-temperature sintering to form CaO, while subsequent reactions with ambient moisture led to the formation of Ca(OH)₂.

### Mechanical properties of Zn-3Mg/xSnS composites

**Fig. 5:**
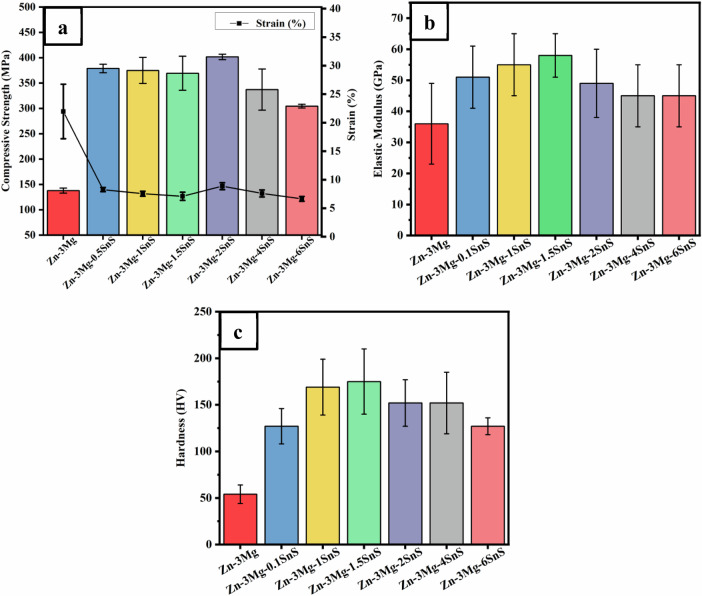
Influence of %wt SnS particulates on **a** compressive strength vs strain **b** Elastic modulus **c** Hardness

The mechanical characterization of the Zn-3Mg/SnS composites was evaluated by means of compressive strength and elastic modulus (Fig. 5a, b) and microhardness (Fig. 5c) results. Among all samples, the Zn-3Mg alloy exhibited the lowest value for these properties, which points to its limited load-bearing capability. On the other hand, the Zn-3Mg alloy showed a relatively higher strain index, suggesting higher ductility compared to the composites (Fig. 5a). The relatively lower mechanical performance of the Zn-3Mg was attributed to the weak interfacial bonding between zinc and magnesium phases, along with the presence of coarse grains (Fig. 3) [40]. Compressive strength, elastic modulus, and hardness, in contrast, were all markedly enhanced by the addition of SnS particles, with values rising in direct proportion to the SnS content up to a certain limit (Fig. 5a–c). The grain refinement facilitated effective load transfer at the Zn–SnS interfaces. A slight reduction in strength was observed beyond 2 wt% SnS addition, likely due to particle agglomeration or microstructural defects acting as stress concentrators. Overall, these findings demonstrate that controlled SnS reinforcement enhances the mechanical reliability of Zn-based composites, making them more suitable for orthopedic applications where both strength and biocompatibility are required.

With the addition of SnS as reinforcement, compressive strength increased to 433 MPa (191% higher than Zn-3Mg) at 2 wt% SnS (Fig. 5a). The elastic modulus of Zn-Mg-SnS alloys showed a parabolic curve as visible in Fig. 5b. The elastic modulus increased from 36 GPa for Zn-3Mg to 58 GPa for Zn-3Mg-1.5SnS composite (Table 2). While the elastic modulus of Zn-3Mg-2SnS was found to be 47 GPa. This value aligns with the elastic modulus of bone, making it a perfect candidate for orthopedic applications, as it reduces the issue of stress shielding. Parallel to this, the microhardness improved significantly with SnS reinforcement (Fig. 5c). The hardness was 55.3 HV for Zn-3Mg, which increased to 173.5 HV for the 1.5 wt% SnS (214%). The increase in these values is attributed to the grain refinement and the formation of Mg₂Zn₁₁ intermetallics, which transfer load from SnS particles to the matrix. Parallel results with the same strengthening mechanisms have been reported previously for Zn/HA and Mg-Zn/HA composites [2, 41]. When the amount of reinforcement increased beyond 2 wt%, compressive strength decreased (minimum 304.4 MPa at 6 wt% SnS), because of agglomeration, coarser intermetallics, and porosity (Fig. 3). This behavior was also parallel to the previous studies [2, 23]. A slight reduction in the hardness results was also observed beyond 1.5 wt% SnS, which is attributable to porosity, consistent with SEM results (Fig. 3f, g).

**Table 2:** Mechanical properties of Zn-Mg-SnS composites

Sr #	Composition	Compressive strength (MPa)	% Strain	Elastic Modulus (GPa)	Hardness (HV)
1	Zn-3Mg	137.8 ± 5.08	21.9 ± 4.78	36 ± 23	54 ± 10
2	Zn-3Mg-0.5SnS	378.8 ± 8.50	8.26 ± 0.36	52 ± 18	127 ± 19
3	Zn-3Mg-1SnS	374.9 ± 25.6	7.53 ± 0.43	55 ± 21	169 ± 30
4	Zn-3Mg-1.5SnS	369.3 ± 33.5	7.09 ± 0.71	58 ± 16	175 ± 35
5	Zn-3Mg-2SnS	401.6 ± 5.43	8.87 ± 0.60	47 ± 20	152 ± 25
6	Zn-3Mg-4SnS	337.1 ± 40.6	7.58 ± 0.62	45 ± 18	152 ± 33
7	Zn-3Mg-6SnS	304.4 ± 3.66	6.64 ± 0.41	45 ± 18	127 ± 9

From the biomechanical side, the compressive strength of all Zn-3Mg/SnS composites exceeded that of human cortical bone (130–240 MPa) [42]. The lowest mechanical performance was observed in 6 wt% SnS sample, which is still 27% stronger than bone, while the optimum composite, 2 wt% SnS, was 67% stronger. These obtained results highlight the usability of Zn-3Mg/SnS composites as a new biodegradable load-bearing orthopedic implants, with balanced strength, stiffness, and biocompatibility.

### Degradation studies

**Fig. 6:**
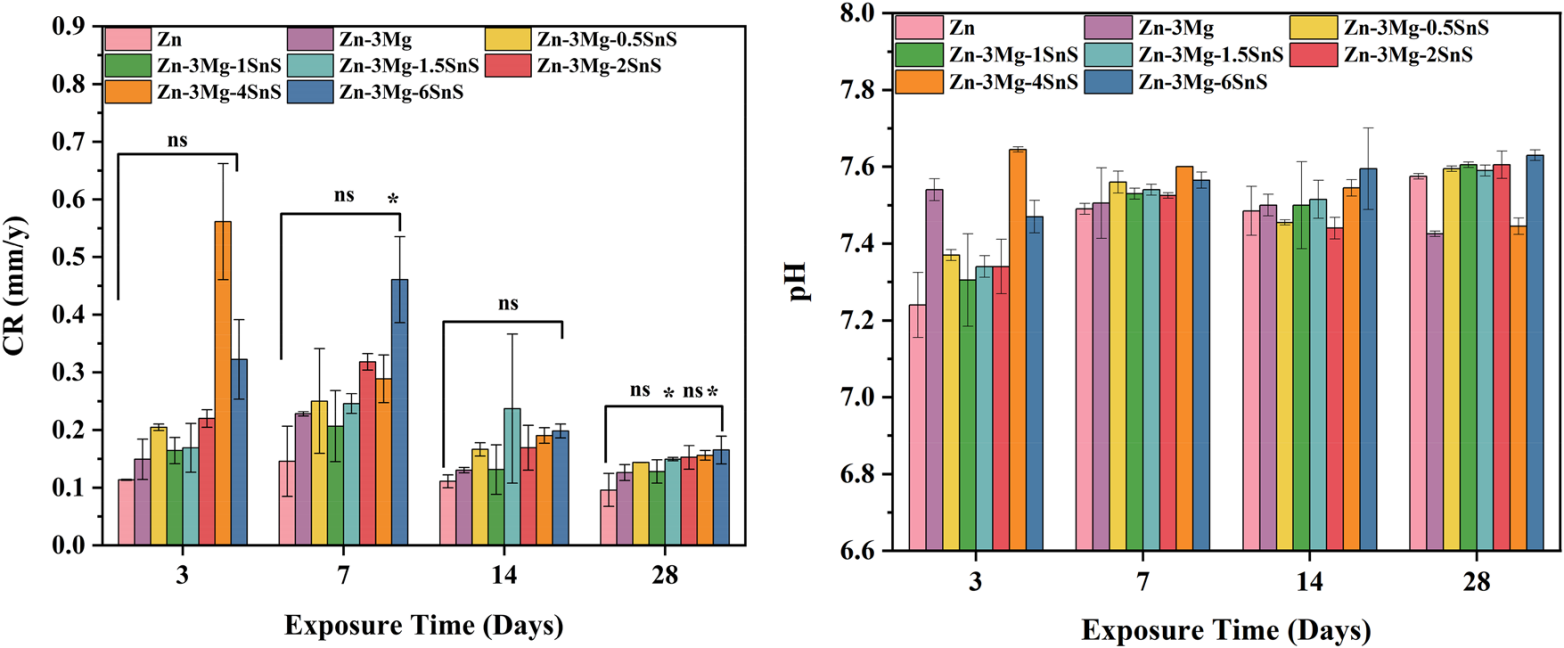
Corrosion rate (left) and pH (Right) of Zn-Mg-SnS composites in SBF

The corrosion behaviour of pure Zn and Zn-Mg-SnS composites in SBF is shown in Fig. 6. The lowest corrosion rate was observed on the third day for the pure Zn samples with 0.11 mm/y (Table 3), whereas Zn-3Mg-0.5SnS showed a slightly higher rate of 0.20 mm/y. This value was 0.32 mm/y for the Zn-3Mg-6SnS. The higher initial corrosion rate can be attributed to the microstructural modifications introduced by SnS addition, the effect of secondary phases that promote localized dissolution.

**Table 3:** Corrosion rates of Zn-Mg-SnS composites calculated from weight loss

Sr #	Composition	Corrosion rate (mm/y)			
		Day 3	Day 7	Day 14	Day 28
1	Zn	0.1136 ± 0.0008	0.1458 ± 0.060	0.1111 ± 0.011	0.096 ± 0.028
2	Zn-3Mg	0.1494 ± 0.035	0.2280 ± 0.003	0.1304 ± 0.004	0.1262 ± 0.013
3	Zn-3Mg-0.5SnS	0.2048 ± 0.005	0.2502 ± 0.090	0.1665 ± 0.011	0.1437 ± 0.0001
4	Zn-3Mg-1SnS	0.1643 ± 0.022	0.2067 ± 0.061	0.1314 ± 0.043	0.12807 ± 0.020
5	Zn-3Mg-1.5SnS	0.1691 ± 0.042	0.2457 ± 0.017	0.2372 ± 0.129	0.1494 ± 0.002
6	Zn-3Mg-2SnS	0.2202 ± 0.015	0.3180 ± 0.014	0.1693 ± 0.038	0.1526 ± 0.020
7	Zn-3Mg-4SnS	0.5613 ± 0.153	0.2888 ± 0.041	0.1907 ± 0.013	0.1560 ± 0.008
8	Zn-3Mg-6SnS	0.3225 ± 0.0688	0.461 ± 0.0745	0.1985 ± 0.0119	0.1654 ± 0.0241

As the immersion period increased, the corrosion rate of both pure Zn and Zn-Mg/SnS alloys decreased progressively (Table 3). This result is attributed to the formation of a protective corrosion layer on the alloy surface. The new interface limits direct exposure of the substrate to aggressive chloride and phosphate ions in SBF. This passive layer has been previously observed in zinc-based biodegradable materials, where rapid ion release occurs during early immersion, followed by stabilization as protective films develop [42].

This controlled degradation rate is highly beneficial for biomedical applications. Because the initial release of Zn²⁺ (and trace Ca²⁺ detected from secondary phases) can stimulate osteogenesis and antibacterial activity, while the subsequent slowdown in corrosion helps preserve mechanical integrity during the critical bone-healing period. These findings demonstrate that Zn-Mg-SnS composites combine biofunctionality with appropriate degradation behavior, making them promising candidates for load-bearing biodegradable implants [42].

During the immersion tests of the Zn-Mg-SnS composites, the pH change was observed for 28 days (Fig. 6) to understand the effect of corrosion products. In the beginning of the tests, a slow rise in pH was observed, which is because of the alkaline corrosion products like Ca₃(PO₄)₂ that were formed as a result of the release of Zn²⁺, Mg²⁺, and trace Ca²⁺ ions and consumption of H+ ions. Furthermore, Ca^2+^ could precipitate as compounds resembling hydroxyapatite in the presence of phosphate ions, further buffering the solution. As the production and dissolution of corrosion products got closer to equilibrium, the pH stabilized with extended immersion.

During this period, the observed pH values remained within the biologically acceptable range (6.5–8.5), as recommended by ISO 10993-5 for in vitro cytocompatibility testing. For biomedical applications, this mild alkalization is beneficial because it inhibits the sharp pH increase that is frequently observed previously in Mg-based implants [43] while also encouraging the deposition of hydroxyapatite, which improves osteointegration and bone bonding.

**Fig. 7:**
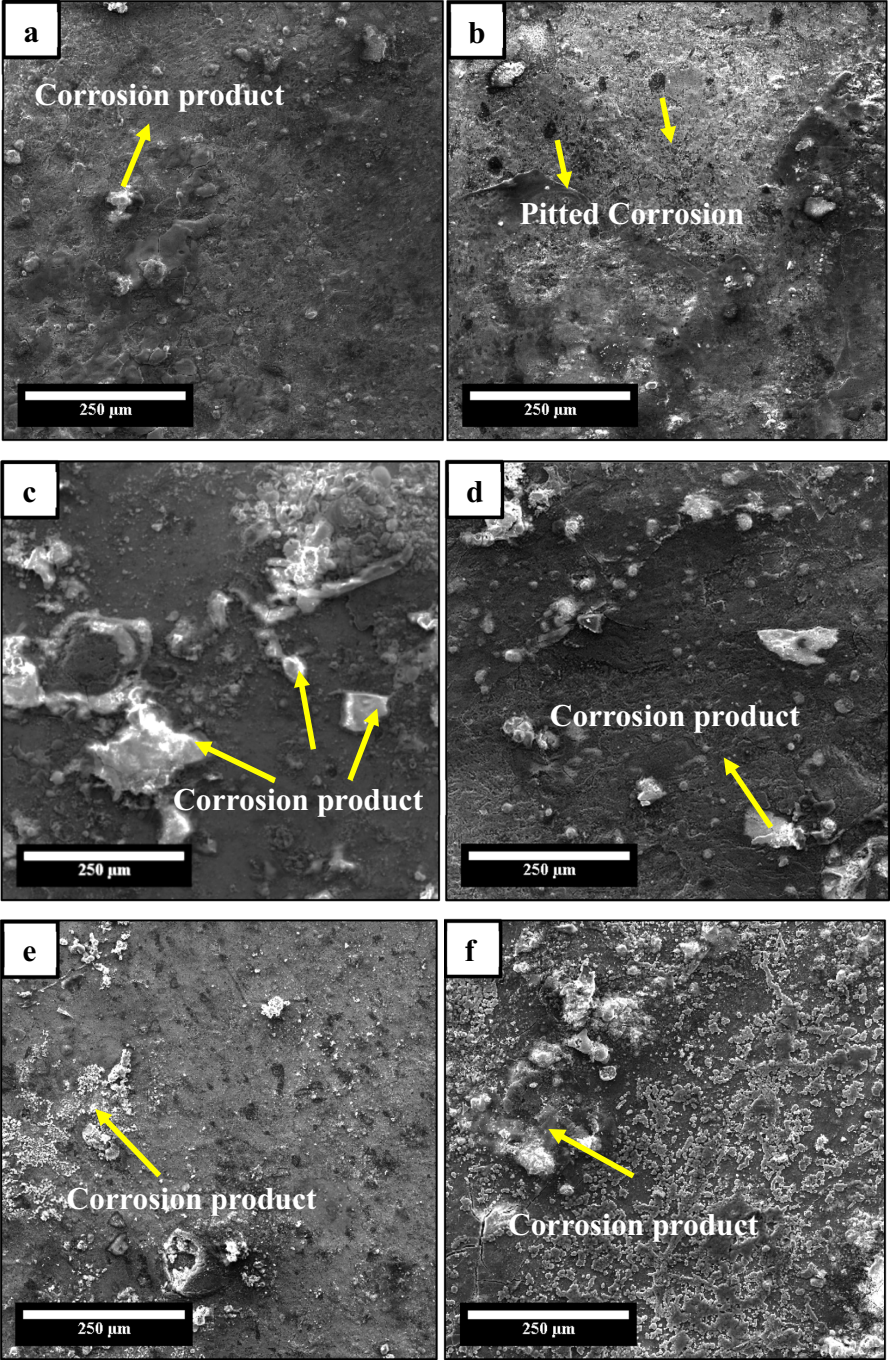
SEM (SE) of **a** Zn-3Mg-0.5SnS **b** Zn-3Mg-1SnS **c** Zn-3Mg-1.5SnS **d** Zn-3Mg-2SnS **e** Zn-3Mg-4SnS **f** Zn-3Mg-6SnS

The surface morphologies of the composites after immersion test in SBF for 672 h are shown in Fig. 7. As seen in Fig. 3, the as-sintered composite surface appeared relatively smooth. After degradation, the SEM images revealed a rough and heterogeneous morphology. This surface roughness is a natural result of corrosion, where electrochemical reactions form corrosion layers. Due to this mechanism, a thin, continuous passive film formed across certain regions of the degraded surface. The grey areas are likely composed of corrosion products formed through interactions between the alloy and ionic species in SBF (Fig. 7a, c, e). These products act as a barrier, inhibit ion exchange, and thereby reduce the overall corrosion rate. Moreover, in some regions, pitting corrosion was observed with localized pores (Fig. 7b). However, these sites were surrounded by more extensive passive film formation, implying that although degradation initiated locally, the subsequent growth of corrosion products effectively slowed further attacks. Importantly, calcium and phosphate ions from SBF may also be incorporated into the passive layer, which would promote the precipitation of phases that resemble hydroxyapatite. It is well known that these bioactive surface coatings improve osseointegration and bone bonding, which is beneficial for orthopedic applications [44].

### Cytocompatibility assessment of Zn-3Mg/xSnS composite

**Fig. 8:**
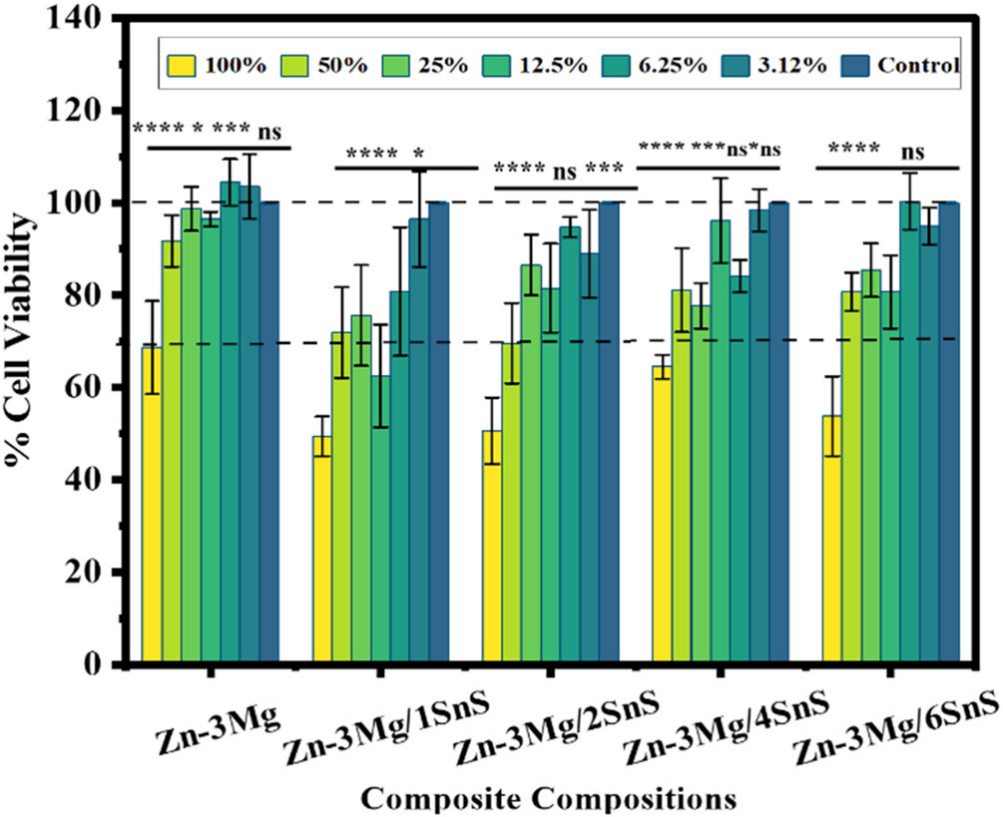
Cell Viability of L929 Fibroblast cells at different extract concentrations

Figure 8 illustrates the viability of L929 fibroblast cells cultured in varying concentrations (100%, 50%, 25%, 12.5%, 6.25%, and 3.12%) of Zn-3Mg and Zn-3Mg–SnS extract solutions. For Zn-3Mg, cell viability in the 100% extract was 70%, which is generally considered the threshold for cytocompatibility in vitro. In contrast, cells cultured in 100% extracts of Zn-3Mg–xSnS composites demonstrated viability values below this threshold, indicating cytotoxicity. When the extract concentration was reduced to 50%, all Zn-3Mg–xSnS composites exhibited a significant increase in cell viability ( ≥ 70%). This positive trend continued with further dilution (25%, 12.5%, 6.25%, and 3.12%), confirming that lower extract concentrations favor cellular proliferation. Importantly, none of the tested materials exhibited cell viability below 50% at any concentration, which indicates the absence of severe cytotoxic effects.

The enhanced cellular response of the SnS-containing composites was parallel to the corrosion studies, which found that the development of protective surface layers moderates release into the medium. A more favorable biological environment for cell survival and proliferation was probably supported by the controlled release of Zn^2+^ and Mg^2+,^ which prevented excessive ion accumulation.

Overall, these results suggest that both Zn-3Mg and Zn-3Mg–SnS composites possess acceptable cytocompatibility, particularly at extract concentrations of 50% or lower. It is possible that the SnS-doped alloys’ improved performance results from their moderated ion release, which avoids excessive Zn^2+^ accumulation that might otherwise hinder cell growth. It should also be noted that these in vitro cytotoxicity tests were performed in a static culture environment, which provides preliminary insights into cell–material interactions. However, this system does not fully replicate the dynamic and regulated conditions of the human body, where ion clearance and protein-mediated buffering occur under an open-loop physiological principle [45–48].

The Zn-3Mg alloy and Zn-3Mg/SnS composites observed cytocompatibility are in line with earlier findings [34, 49–52]. Watroba et al. [50] investigated Zn alloys and observed similar cytotoxic effects at higher extract concentrations (100%, 50%, and 25%). On the other hand, at lower dilutions (12.5% and 5%), the alloys exhibited good cell viability, which means non-cytotoxic behaviour. Similar results were reported by Tang et al. and Danbatta et al. [50, 51] for Zn–xCu alloys (*x* = 1–4 wt.%). According to their obtained results, extracts at 50% and 10% concentrations were well tolerated by EA.hy926 cells, whereas 100% extracts induced cytotoxicity. Higher Zn²⁺ levels in the culture media were the reason for this reaction at higher concentrations, according to Danbatta et al. [35, 52]. Parallel to these observations, the current study shows that adding SnS to Zn-3Mg considerably increases cytocompatibility at higher extract concentrations, indicating that it may be a safer and more efficient zinc-based material for orthopedic applications.

## Conclusion

In this experimental study, Zn-3Mg alloys reinforced with 0.5–6 wt% SnS composites particulates were successfully manufactured. The obtained composites exhibited a lightweight structure with densification rates of 86.3–97.2%. The increase in SnS reinforcement decreased the sinterability index of the composite. According to the mechanical characterization results, the Zn-3Mg/2SnS composite was the best combination of properties, with a compressive yield strength of 402 MPa, elastic modulus of 49 GPa, and hardness of 151 HV. These improvements in the material properties were attributed to the homogeneous distribution of SnS reinforcements and fine intermetallic phase formation in the matrix. Cytocompatibility tests verified non-toxicity at extract concentrations of 50% or less, while degradation test results showed a corrosion rate of 0.2 mm/year for 28 days. According to these obtained results, these composites are promising candidates for the new generation of biodegradable materials for orthopedic applications.

## Abbreviations

ANOVA: Analysis of Variance
BMMCs: Biodegradable Metal Matrix Composites
BZMCs: Biodegradable Zinc Matrix Composites
CR: Corrosion Rate
CYS: Compressive Yield Strength
HA: Hydroxyapatite
PM: Powder Metallurgy
SnS: Sintered Snail Shell
SPS: Spark Plasma Sintering
SEM: Scanning Electron Microscopy
SBF: Simulated Body Fluid
XRD: X-ray Diffraction
UTS: Ultimate Tensile Strength
MTT: 3-(4,5-dimethylthiazol-2-yl)-2,5-diphenyltetrazolium bromide
